# Impact of emotional and motivational regulation on putting performance: a frontal alpha asymmetry study

**DOI:** 10.7717/peerj.6777

**Published:** 2019-04-26

**Authors:** Tai-Ting Chen, Kuo-Pin Wang, Ming-Yang Cheng, Yi-Ting Chang, Chung-Ju Huang, Tsung-Min Hung

**Affiliations:** 1 Departement of Physical Education, National Taiwan Normal University, Taipei, Taiwan; 2 Faculty of Psychology and Sport Science, Universität Bielefeld, Bielefeld, Germany; 3 Graduate Institute of Sport Pedagogy, University of Taipei, Taipei, Taiwan; 4 Department of Physical Education & Institute for Research Excellence in Learning Science, National Taiwan Normal University, Taipei, Taiwan

**Keywords:** Golf, Anxiety, Attention, Self-regulation

## Abstract

**Background:**

The efficacy of emotional and motivational regulation can determine athletic performance. Giving the short duration and fast changing nature of emotions experienced by athletes in competition, it is important to examine the temporal dynamics of emotional and motivational regulation. The aim of this study was to investigate emotional and motivational regulation as measured by frontal alpha asymmetry in skilled golfers during putting performance after a performance failure.

**Methods:**

Twenty skilled university golfers were recruited and requested to perform 40 putts at an individualized difficulty level of 40–60% successful putting rate. Trials immediately after a failed putt were selected for analysis. Successful performances were those trials where a hole was and unsuccessful performances were those that failed. The frontal alpha asymmetry index of LnF4-LnF3 was derived for statistical analysis.

**Results:**

(1) Successful performance was preceded by a larger frontal alpha asymmetry index at T2 than that of T1, and (2) a larger frontal alpha asymmetry index was observed for unsuccessful performance than for successful performance at T1.

**Discussion:**

The results suggest that successful emotional and motivational regulation was characterized by a progressive increase of frontal alpha asymmetry, which led to subsequent putting success when facing an emotionally provocative putting failure. These findings shed light on the application of frontal alpha asymmetry for the understanding and enhancement of emotional and motivational regulation during sport performance.

## Introduction

“Emotional regulation” is a term generally used to describe a person’s ability to use strategies to initiate, maintain, modify, or display emotions (Gross & Thompson, 2007). Emotions play important roles, as they ready necessary behavioral responses, tune decision making, enhance memory for important events, and facilitate interpersonal interactions (Gross & Thompson, 2007). Athletes constantly attempt to regulate emotions if they believe that doing so will facilitate performance (Lane et al., 2012). For example, an athlete might seek advice from a coach, take a deep breath, and visualize successful outcomes to reduce their anxiety to regain the feelings associated with winning. On the contrary, when athletes make a mistake during a game, they might subsequently suffer from anxiety arising from fear of making the same mistake. If they do not have the ability to regulate that emotion, their performance worsens. Therefore, the efficacy of emotional regulation may be a key differentiating factor for elite athletes.

The depletion of emotional self-regulation resources influences performance. Wagstaff (2014) showed that compared with a control group (that received no video treatment) and a nonsuppression group (that was given no self-regulation instructions during video watching), participants who suppressed their emotional reactions to an upsetting video completed a 10-km cycling task more slowly, generated lower mean power outputs, reached a lower maximum heart rate, and perceived greater physical exertion. The findings suggest that excessive suppression of emotion may deplete self-regulation resources (Baumeister, Vohs & Tice, 2007), which subsequently results in impaired physical performance and increased mental fatigue. Although past studies have used films (Dennis & Solomon, 2010), pictures (Pérez-Edgar et al., 2013), or words (Kessler et al., 2009) with negative, neural, or positive valence as emotional stimuli, which are effective at inducing emotion, emotional induction from stimuli that are frequently encountered by athletes during real life settings would increase the ecological validity of these findings in the sport context.

Failure is common in sport performance, and it is also one of the most emotionally laden stimuli during competition. When a failure occurs, there is still little known about how the athlete regulates their emotions, which affects subsequent performance. Moreover, emotion is a state with relatively short duration. It has been suggested that the few seconds prior to skill execution is critical for subsequent sport performance (Kao, Huang & Hung, 2013). As the emotional state during competition can fluctuate as a result of various environmental and psycho-social influences, it is important to understand the dynamic nature of emotional regulation right before performance start.

Questionnaires have been widely used for assessing emotional state in sports. However, they are of limited use in assessing the fast fluctuating emotional states during the short pre-performance period. Alternatively, psychophysiological measurement such as electroencephalography (EEG) can be used to ameliorate this limitation. In addition to the strength of high temporal resolution (milliseconds), which is suitable for capturing the dynamic nature of mental state prior to performance, several EEG components have been associated with emotion. For example, delta (Meerwijk, Ford & Weiss, 2015), theta (Uusberg, Thiruchselvam & Gross, 2014), alpha (Quaedflieg et al., 2015), beta (Morillas-Romero et al., 2015), and gamma (Balconia & Lucchiari, 2008) in the frontal area have been implicated in affective states. Among these measurements, the frontal alpha asymmetry has been suggested for assessing emotional regulation. Frontal alpha asymmetry is the measure of differences in alpha frequency (8–12 Hz) band power between the left (F3) and right (F4) side of the frontal lobe (Hugdahl & Davidson, 2003). Alpha power represents the inverse of cortical activation (Klimesch, Sauseng & Hanslmayr, 2007). According to the valence hypothesis (Tomarken et al., 1992), positive emotions are associated with higher left prefrontal cortex activation (e.g., lower alpha power) whereas negative emotions are related to higher right prefrontal cortex activation. Papousek et al. (2014) showed that reduced alpha at right compared to left frontal lobe was associated with negative emotions (sadness) after unpleasant film viewing, whereas reduced alpha at left compared to right frontal lobe was accompanied by positive emotion (pleasantness) after positive film viewing (Wheeler, Davidson & Tomarken, 1993). Moreover, Dennis & Solomon (2010) suggested that frontal alpha asymmetry is also observed in event-related stimuli, which might reflect the ability to regulate emotions in specific contexts. In addition to the valence hypothesis, Harmon-Jones & Gable (2017) maintained that frontal alpha asymmetry reflects increased approach motivation. Relatively increased frontal asymmetry (i.e., higher alpha power in the right relative to the left frontal lobe) may serve as approach motivation or related emotion. In contrast, relatively decreased frontal asymmetry may serve as withdrawal motivation. Evidently, frontal alpha asymmetry activity is not only a reflection of positive/negative emotions, but also an ability to regulate functional approach/withdrawal motivation and emotion depending on the demands of the situation.

A recently developed model for exploring the relationship between emotional regulation and optimal performance is the multi-action plan model (MAP model; Robazza et al., 2016). MAP depicts four performance categories, specifically, optimal-automated (Type 1), optimal-controlled (Type 2), sub optimal-controlled (Type 3), and suboptimal-automated (Type 4), derived from a hypothesized interaction of optimal/suboptimal and automatic/controlled performance dimensions. The MAP model could serve as a useful theoretical framework for the examination of emotional regulation prior to motor performance (Fronso et al., 2017). In particular, when an athlete is facing a difficult task (e.g., a distance of 40–60% holed rate), they need to exert conscious effort to focus on the individual’s core components of action to “control” performance. This is particularly challenging after a previous putting failure. If the athlete can regulate the emotion that leads to the following successful putting performance, they are considered to be in a Type 2 state. On the contrary, if the subsequent performance failed, they are in a Type 3 state.

Giving the scant research regarding emotional regulation during sport performance and the potential of frontal alpha asymmetry measurement for exploring this issue, the purpose of this study was to examine the temporal dynamic of frontal alpha asymmetry with an ecologically relevant emotional stimuli (i.e., facing failure). We hypothesized that after failed putting, subsequent successful putting would be preceded by progressively reduced alpha power in the left compared to the right frontal lobe, whereas subsequent unsuccessful putting would be preceded by progressively reduced alpha power in the right compared to the left frontal lobe.

## Materials & Methods

### Participants

Sixteen male and four female skilled golfers (all right handed) ranging in age from 18 to 29 (mean age = 20.33 ± 2.54) volunteered to participate in the study. They practiced at least five times a week and often participated in national or international competitions (mean year = 7.70 ± 2.41, mean best handicap =−2.95 ± 2.42). Moreover, participants were screened with a health history questionnaire to ensure that all were free of neurological disorders or not taking any related medicine. All participants were asked to read and sign an informed consent form and were free to withdraw from the study at any time. The task had no physical or mental risk. To respect privacy, the information and data from participants were processed with confidentiality and anonymity. This study was approved by the institutional review board of National Taiwan University (NTU-REC No.: 201312ES055).

### EEG recording

In accordance with the international 10–20 system, electrode sites of brain waves were recorded on F3, F4, C3, C4, P3, P4, T3, T4, O1 and O2. Electrical reference was located on the left and right ear mastoids (A1, A2), and the ground electrode was located at FPz. Vertical and horizontal electrooculograms were recorded with bipolar configurations located superior and inferior to the left eye and on the left and right orbital canthi. Neuroscan software 4.3 was used to collect data, with a band pass filter setting from DC to 100 Hz. A 60 Hz Notch filter was kept on during the data recording, and the sampling frequency was 500 Hz. Electrode impedance was kept below 5 kΩ.

### Putting task

Forty straight putts were taken to a regulation hole (10.80 cm diameter) on an artificial putting green (length = 7 m, width = 0.9 m) and the putting distance was determined based on a 40–60% successful putting rate. All participants used their own golf putters and standard size (4.27 cm diameter) white golf balls. The putting performance subsequent to a failed putt was the trial of interest. These trials of interest were classified into successful and unsuccessful performances depending on whether they were putted into the hole or not.

### Procedure

All participants were asked to refrain from drinking coffee and alcoholic beverages the day before testing. Next, they were informed of their right to withdraw from the study at any time during the data collection process, and then provided informed consent. They were fitted with a Lycra electrode cap (Quick-cap; Neuroscan, Charlotte, NC, USA). Electroencephalogram signals and resistance were checked. Participants attempted to keep their eyes open without blinking and kept their body stable for at least 2 s before the backswing. Putting distance was designated 40–60% putting success rate and 300 centimeters was the beginning distance they putted. They performed 10 putts and the distance was adjusted relying on whether the average of 10 putting success rate was 40–60% or not. If the success rate was between 40 to 60%, then the putting distance was set at 300 cm. If the success rate was above 60%, then the putting distance would increase 30 cm and then they performed extra 10 putts until the success rate reached 40–60%. On the contrary, if the success rate was below 40%, then the putting distance would decrease 30 cm and then they performed extra 10 putts until the success rate reached 40–60%. After the appropriate putting distance was decided, the participant performed 40 putts to the best of their ability. Each block encompassed 10 putts and participants could rest for 2 min between blocks. In each trial the backswing movement was detected by an infrared sensor as an event marker. To increase the ecological validity of the experiment, participants were instructed to maintain their routine and fix their eyes on the ball during the last two seconds before putting action. The balls were prepared and retrieved by the experimenter for the golfers after each putt so that the green was clear from any putted balls.

### Data analysis

Neuroscan Edition 4.3 software was used to perform offline EEG data processing such as baseline correction, EOG correction, and artifact rejection. Ocular artifacts such as eye blinks and lateral eye movements were automatically removed using regression methods (Gratton, Coles & Donchin, 1983) and other artifacts were visually checked and manually rejected. To reduce interference from the participants’ pre-performance routine and preparatory tempo and increasing ecological validity, they were told to keep their bodies static and their eyes on the ball for at least 2 s before the backswing. To analyze the pre-putt emotional state, at least 2 s before the backswing was needed for each trial. Each 2-s epoch was baseline-corrected based on the entire sweep and was then segmented into two 1-s epochs (T1:-2- 1 s. and T2:-1- 0 s.). The baseline correction was performed after excluding those trials with amplitude exceeding ±100 mv. In addition, band-pass filter was set at 1–30 Hz with a slope of 12 db/oct. EEG were processed through the fast Fourier transform with Hanning window. 8–12 Hz was selected for the alpha frequency band. The averaged alpha power (µV^2^) at F3 and F4 sites was derived. Asymmetry score = ln (F4 alpha power)–ln (F3 alpha power).

### Statistical analysis

With a sample of 20 participants, an alpha of 0.05, and power = 0.8, a sensitivity analysis was conducted, which showed that sample size was powered to detect a medium to large effect size ES = 0.66. Kolmogorov–Smirnov was used to do the test of normality. The result was 0.2, larger than 0.05; the data could thus be considered normally distributed. Additionally, the equation of emotional regulation was based on simple models used in previous EEG Asymmetry research. Specifically, the Frontal alpha asymmetry index was obtained by applying the equation of ln (F4) - ln (F3) (Jaworska et al., 2012). Because alpha power is often interpreted as inversely related to cortical activity, higher values on this index reflect greater left frontal activation and lower values reflect greater right frontal activation (Allen, Coan & Nazarian, 2004). Two-way repeated measures analysis of variance 2 (performance: successful, unsuccessful) ×2 (time: T1, T2) was applied to the Frontal alpha asymmetry index. When interaction occurred, simple main effects were tested. Alpha was set to .05 for all analyses, and effect sizes were calculated using partial eta squared ($\eta { }_{p}^{2}$).

## Results

### Putting performance

Participants missed 26.50 ± 4.86 putts at the 40–60% difficulty level. Following these misses, participants made the next putt 8.1 ± 2.27 times, on average; and missed their next putt 18.4 ± 5.92 times on average.

### Frontal alpha asymmetry

**Figure 1:**
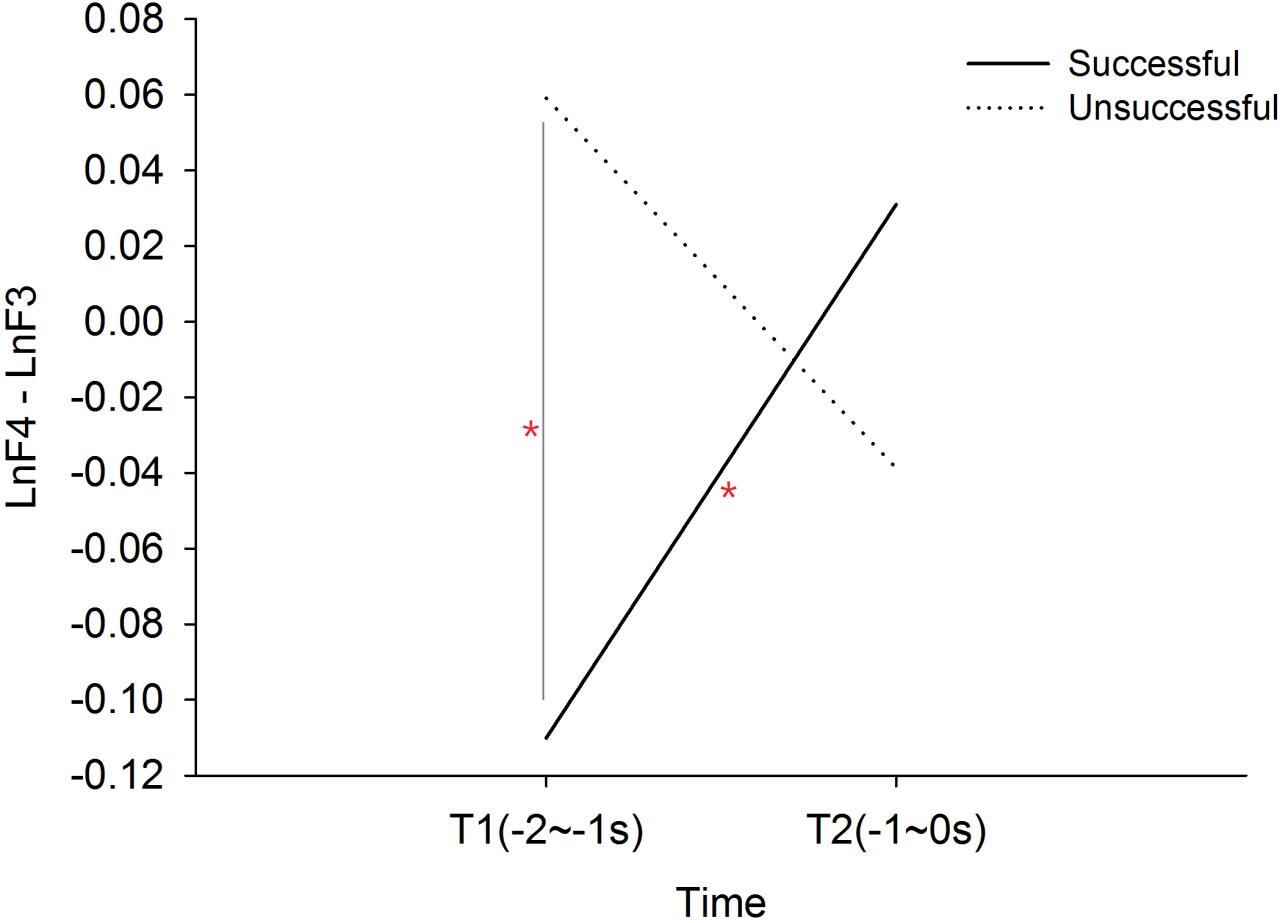
Frontal alpha asymmetry between successful and unsuccessful performance at T1 (-2∼-1s) and T2 (-1∼0s).

The 2 × 2 (performance × time) ANOVA revealed a significant performance × time interaction effect, F(1, 19) = 14.583, *p* = .001, $\eta { }_{p}^{2}=.434$. The post hoc simple main effect analysis showed that successful performance was preceded by a progressively increased frontal alpha asymmetry index T1 to T2, *t*(19) = 2.420, *p* = .026, Cohen’s *d* = .733. Main effect of performance is *F*(1, 19) = 2.469, *p* = .133, $\eta { }_{p}^{2}=.115$; time *F*(1, 19) = .194, *p* = .664, $\eta { }_{p}^{2}=.010$. Mean frontal alpha asymmetry of successful performance is -.40; Mean frontal alpha asymmetry of unsuccessful performance is .010. In addition, the frontal alpha asymmetry index for successful performance was smaller than unsuccessful performance at T1, *t*(19) =  − 3.920, *p* = .001, Cohen’s *d* = .798 (See Fig. 1). There were no performance or time main effects.

### Control analysis

Three additional analyses were performed to provide more support for using the frontal alpha asymmetry index as a measure of emotion.

#### Regional specificity of frontal alpha asymmetry index

In order to demonstrate that the alpha asymmetry index is specific to the frontal region, alpha asymmetry indices were computed for the central, parietal, temporal, and occipital regions. Several performance X time two-way ANOVAs were employed to separately assess the alpha asymmetry index in central, temporal, parietal, and occipital regions. Results showed that neither performance main effects (central region, *F*(1, 19) = .000, *p* = .983, $\eta { }_{p}^{2}=.000$; parietal region, *F*(1, 19) = .021, *p* = .885, $\eta { }_{p}^{2}=.001$; occipital region, *F*(1, 19) = .004, *p* = .948, $\eta { }_{p}^{2}=.000$; temporal region, *F*(1, 19) = .199, *p* = .660, $\eta { }_{p}^{2}=.01$), nor interaction effects (central region, *F*(1, 19) = 3.573, *p* = .074, $\eta { }_{p}^{2}=.158$; parietal region, *F*(1, 19) = .788, *p* = .386, $\eta { }_{p}^{2}=.040$; occipital region, *F*(1, 19) = 1.408, *p* = .250, $\eta { }_{p}^{2}=.069$; temporal region, *F*(1, 19) = 2.447, *p* = .134, $\eta { }_{p}^{2}=.114$) were significant.

#### Frequency specificity of frontal alpha asymmetry index

The alpha band in the frontal area was the dominant frequency band related to putting performance. Neighboring frequencies of alpha in the frontal region were examined. Specifically, the frequency bands of 4–8 Hz and 12–16 Hz were subjected to a performance X time two-way ANOVA separately. Results showed there were neither interaction effects in 4–8 Hz, *F*(1, 19) = 3.919, *p* = .062, $\eta { }_{p}^{2}=.171$, and 12–16 Hz, *F*(1, 19) = .609, *p* = .445, $\eta { }_{p}^{2}=.031$, nor performance main effects in 4–8 Hz, *F*(1, 19) = .540, *p* = .471, $\eta { }_{p}^{2}=.028$, and 12–16 Hz, *F*(1, 19) = .262, *p* = .614, $\eta { }_{p}^{2}=.014$.

#### Equality of baseline frontal alpha asymmetry

The association of frontal alpha asymmetry during putting with putting performance can be further supported by ruling out the inequality of frontal alpha asymmetry at baseline. The one-sample *t* test, *t*(19) = 1.094, *p* = .288, showed that participants were in the emotionally neutral state (mean = 40.04 ± 0.16) before the putting task began.

#### Alpha power analysis

Alpha power analysis was performed to determine the hemispheric contribution to the frontal asymmetry observed during the preparatory period right after a failed putt. A 2 × 2 × 2 Performance (successful, unsuccessful) × Time (T1, T2) ×Hemi (Left, Right) ANOVA showed a significant Performance X Time X Hemi interaction effect, *F*(1, 19) = 16.118, *p* = .001, $\eta { }_{p}^{2}=.446$. The follow up simple interaction effect analysis revealed that

**Table 1:** Alpha power analysis.

PP	CP	HEMI	TIME	Ln (ALPHA POWER)
S	S	F3	T1	1.086 ± .121
			T2	1.324 ± .097
		F4	T1	1.108 ± .124
			T2	1.212 ± .105
	U	F3	T1	.935 ± .123
			T2	1.245 ± .125
		F4	T1	.897 ± .121
			T2	1.282 ± .123
U	S	F3	T1	1.341 ± .100^*^
			T2	1.027 ± .125^*^
		F4	T1	1.231 ± .109^*^
			T2	1.058 ± .124^*^
	U	F3	T1	1.264 ± .128^*^
			T2	.935 ± .123^*^
		F4	T1	1.323 ± .132^*^
			T2	.897 ± .121^*^

**Notes.**

* Significant at < 0.05.

PP prior putt performance CP current putt performance Hemi Hemisphere S successful putt U unsuccessful putt

1. On successful performance condition, the Time main effect, *F*(1, 19) = 10.405, *p* = .004, $\eta { }_{p}^{2}=.354$, and the Hemi X Time interaction effect, *F*(1, 19) = 5.859, *p* = .026, $\eta { }_{p}^{2}=.236$ were significant. Given this interaction effect, a subsequent simple main effect analysis was performed and showed a progressive reduction of alpha power from T1 to T2, *t*(19) = 4.272, *p* = .000, Cohen’s *d* = 2.774, in the left hemisphere, and at T1 alpha power was significantly lower in the right compared to the left hemisphere, *t*(19) = 2.383, *p* = .028, Cohen’s *d* = 1.052. However, in unsuccessful performance conditions, only the Time main effect, *F*(1, 19) = 35.661, *p* = .000, $\eta { }_{p}^{2}=.652$, reached significance (See Table 1).
2. In the left hemisphere, the result showed the Time main effect *F*(1, 19) = 36.838, *p* = .000, $\eta { }_{p}^{2}=.660$. As for the right hemisphere, both the Time main effect *F*(1, 19) = 17.844, *p* = .000, $\eta { }_{p}^{2}=.484$ and the Performance X Time interaction effects were observed. The subsequent simple main effect analysis demonstrated a progressive reduction of alpha power from T1 to T2, *t*(19) = 5.465, *p* = .000, Cohen’s *d* = 3.364, in unsuccessful performance, whereas at T2 alpha power was significantly lower in unsuccessful performance compared to the successful performance, *t*(19) = 2.879, *p* = .010, Cohen’s *d* = 1.314 (See Table 1).
3. At T1, a Performance X Hemi interaction effect was observed, *F*(1, 19) = 15.369, *p* = .001, $\eta { }_{p}^{2}=.447$. The subsequent simple main effect analysis demonstrated that alpha power was significantly lower in the right compared to the left hemisphere for successful performance, *t*(19) = 2.383, *p* = .028, Cohen’s *d* = 1.052. As for T2, only the Performance main effect was observed, *F*(1, 19) = 6.219, *p* = .022, $\eta { }_{p}^{2}=.247$ (See Table 1).

#### Frontal alpha asymmetry is different between a prior successful and failed putt

**Figure 2:**
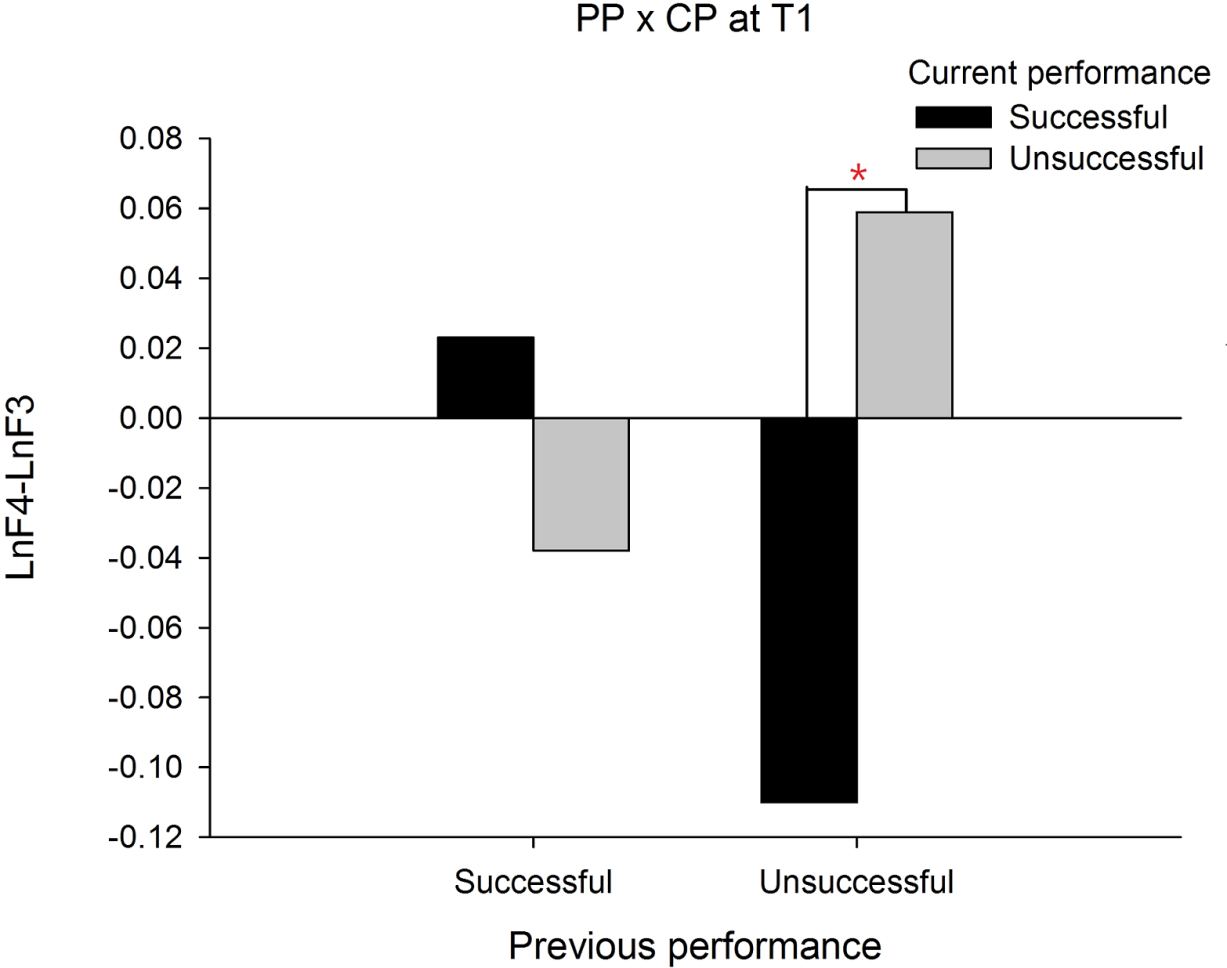
Frontal alpha asymmetry for current successful and unsuccessful performance between previous successful and unsuccessful performance at T1 (-2∼-1s).

**Figure 3:**
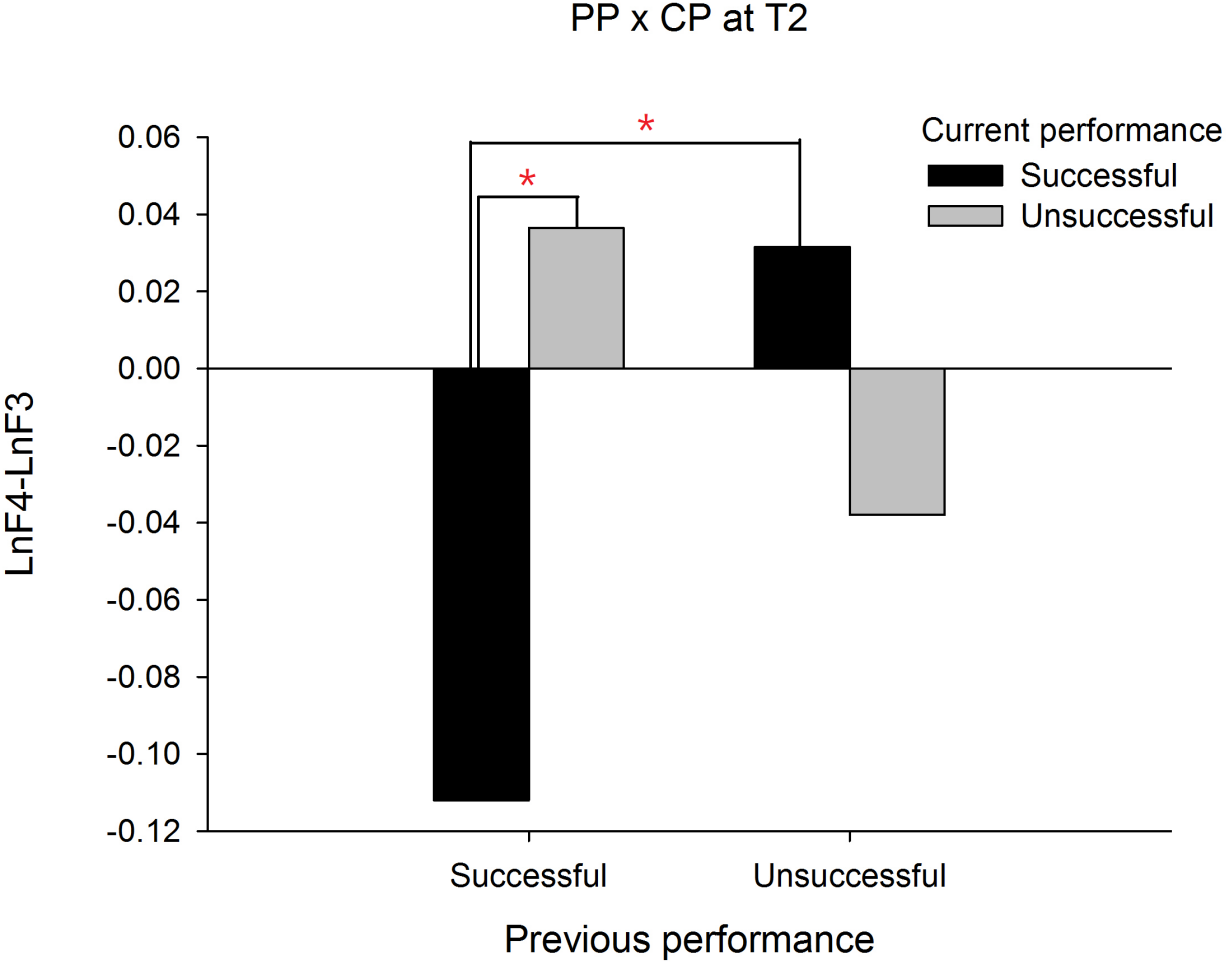
Frontal alpha asymmetry for current successful and unsuccessful performance between previous successful and unsuccessful performance at T2 (-1∼0s).

In order to demonstrate that the frontal alpha asymmetry is unique to a prior failure, we compared the frontal asymmetry patterns between failed and made putts. For this intended purpose, a PP (previous performance) X CP (current performance) X Time three-way ANOVA was performed and we focused only on any effects associated with PP. The results showed a significant interaction effect on PP X CP X Time, *F*(1, 19) = 19.335, *p* = .000, ${\eta }_{p}^{2}=.504$. The follow up simple interaction effect analysis revealed that at T1, a significant PP X CP interaction effect, *F*(1, 19) = 13.140, *p* = .002, ${\eta }_{p}^{2}=.409$ was observed. Subsequent simple main effect analysis demonstrated that the frontal alpha asymmetry in current successful putts was higher than current unsuccessful putts for the previous unsuccessful putt condition, *t*(19) =  − 3.889, *p* = .001, Cohen’s *d* = 0.794 (See Fig. 2). Similarly, at T2, the PP X CP interaction effect, *F*(1, 19) = 12.395, *p* = .002, ${\eta }_{p}^{2}=.395$ also reached significance. Subsequent simple main effect analysis demonstrated that the frontal alpha asymmetry in current successful putts was lower than current unsuccessful putts for the previous successful putt condition, *t*(19) =  − 3.085, *p* = .006, Cohen’s *d* = 1.490 (see Fig. 3). On the contrary, the frontal alpha asymmetry in previous successful putts was higher than previous unsuccessful putts for the current successful putt condition, *t*(19) =  − 2.697, *p* = .014, Cohen’s *d* = 0.745. These findings indicate that the frontal alpha asymmetry is different between a prior failure and success.

## Discussion

Frontal alpha asymmetry patterns have been consistently linked to broad patterns of affective style (Davidson, 2004). The current study sought to extend previous work by examining the potential association between frontal alpha asymmetry and sport performance with an intention to demonstrate that frontal alpha asymmetry could also be an indicator for emotional and motivational regulation during competition. We provided real-time psychophysiological evidence in an ecologically valid setting to show that frontal alpha asymmetry was associated with performance in a temporally dynamic manner. Specifically, after failed putts, skilled golfers regulated their emotion and motivation by gradually increasing frontal alpha asymmetry, which resulted in successful performance. On the contrary, the frontal alpha asymmetry was not increased prior to unsuccessful performance. These findings not only provide support to the belief that emotions and motivations influence performance and are consistent to related emotional regulation literature (Totterdell, 2000; Woodman et al., 2009) but also demonstrate that state frontal alpha asymmetry could explain emotional and motivational regulation during emotional challenges (Goodman et al., 2013).

The progressive increase of frontal alpha asymmetry prior to successful performance reflects an increasing activation of the left frontal area. Frontal alpha asymmetry has been associated with emotional states, with increased frontal alpha asymmetry relating to positive emotion whereas reduced frontal alpha asymmetry is linked to negative emotion (Quaedflieg et al., 2016). This finding suggests that when facing a previous putting failure, skilled golfers regulate emotion and motivation by increasing left frontal activity during the last two seconds before putting execution, which results in successful performance. This interpretation was consistent with the alpha power analysis in our control analysis (i.e., control analysis 4). Specifically, on successful performance condition, alpha power progressively reduced from T1 to T2 in the left frontal area, indicating an increase of positive emotional and motivational regulation, which led to successful performance. On the contrary, alpha power progressively decreased from T1 to T2 in the right frontal area on unsuccessful performance condition, indicating an increase of negative emotion. In other words, golfers could use strategies to start, maintain, modify, or display positive emotion (Gross & Thompson, 2007) to cope with the possible negative emotion induced by the failed putt, and perform successfully. There is sufficient evidence to show that athletes can use strategies to create a more appropriate emotional state during competition (Jones, 2012). Negative emotional reactions can exhaust an athlete’s cognitive resources and adversely impact performance if poorly-managed (Tonnaer et al., 2017). On the contrary, if they have better emotional regulation, they can reinforce positive thoughts and feelings (Weytens et al., 2014) and have greater opportunities to win, even in difficult and challenging situations. In contrast, skilled golfers were not able to reverse from previous performance failure when their frontal asymmetry was not increasing during the last two seconds. These findings not only corroborate previous reports of positive emotions enhancing performance (Totterdell, 2000; Uphill, Groom & Jones, 2014; Woodman et al., 2009), but also signify the importance of examining the temporal dynamic of emotional regulation during sport performance, which happens to be the strength of EEG, a high temporal resolution psychophysiological measurement.

The significantly higher frontal alpha asymmetry during the first epoch of motor preparation for failed (following previously failed putts), compared to the successful putts, was unexpected. It implied that emotion at the beginning of the preparatory period in unsuccessful putts situation was relatively positive. According to the viewpoint where frontal alpha asymmetry reflects approach motivation (Harmon-Jones & Allen, 1998), negative emotions such as anger could lead to higher frontal alpha asymmetry. Given that the present study didn’t take any subjective measures of emotion, future studies are encouraged to include subjective emotional measures to precisely pinpoint the exact emotions experienced during the regulatory process preceding successful and unsuccessful performance.

The relevance of frontal alpha asymmetry to sport performance is strengthened by our control analyses. First of all, in order to demonstrate that the frontal alpha asymmetry is unique to a prior failure, we compared the frontal asymmetry patterns between failed and made putts. The results showed that the frontal alpha asymmetry is different between a prior failure and success, which demonstrated the unique requirement for emotional and motivational regulation in the present study. Our control analysis also provided evidence for the regional specificity of frontal alpha asymmetry. It is only in the frontal region, not all other regions including central, temporal, parietal, and occipital regions, that alpha asymmetry was related to putting performance. Similarly, a frequency specificity of frontal alpha asymmetry was supported by showing that only the alpha frequency band, not the neighboring frequency bands in frontal region, was related to putting performance. These two results support the emotional and motivational regulation interpretation of the present study, given the context of facing putting failure during task performance for the skilled golfers. Furthermore, our baseline analysis showed that the golfers were in an emotionally neutral state before the putting task began, which suggested that emotions during putting performance were specifically induced by experimental manipulation.

According to the MAP model, performers regulate their levels of competitive anxiety and pleasant/unpleasant emotions to achieve individualized optimal states for outstanding performance (Robazza, Pellizzari & Hanin, 2004). Bortoli et al. (2012) further specified that Type 2 is hypothesized to be a functional-unpleasant and effortful (nervous, angry) performance state. Our results support this hypothesis by showing that following failure, left frontal cortex was more active from T1 to T2 prior to subsequent successful performance. On the contrary, progressive higher activation from T1 to T2 in the right frontal cortex results in unsuccessful performance. This was consistent with previous studies and offered important information regarding how successful performance in Type 2 was regulated by left frontal activation that accompany with positive emotion and approach related motivation, whereas unsuccessful performance in Type 3 was regulated by right frontal activation that is associated with negative emotion and withdrawal related motivation. In contrast with the automatic processing of Type 1 (functional-pleasant) and Type 4 (dysfunctional-pleasant), Type 2 and 3 are more dependent on the controlled processing as formulated in the MAP model (Bortoli et al., 2012), which could be induced by the prior performance failure used in the present study. Our findings not only provide evidence to support the MAP model, but also go one step further to discriminate Type 2 from Type 3, by showing that the effective and effortful process of Type 2 was characterized by positive-going, emotional and motivational regulation with a very short temporal dynamic nature. The delineation of this refined and dynamic process furthers our understanding of successful regulation. Nevertheless, manipulation of the frontal alpha asymmetry preceding motor performance is needed to establish a causal relationship between emotional and motivational regulation, and subsequent performance.

There are some methodological limitations that should be addressed in future studies. First, intensity and duration are two central characteristics of an emotional response (Brans & Verduyn, 2014). Larger frontal alpha asymmetry index reflects stronger intensity. The assessments were only based on the two seconds prior to putting execution. Giving the advantage of high temporal resolution for EEG measurement, frontal alpha asymmetry measured outside of the two second period could also be important for understanding emotional regulation in skilled golfers. Second, emotions tend to be evoked by certain events, mostly by a cognitive antecedent that determines which emotion is triggered (Schutz & Davis, 2000). Cognitive appraisal plays a key role in determining which emotion was induced (Sakakibara & Endo, 2016). Therefore, subjective measurements of emotion would be useful to clarify exactly what negative or positive emotions are regulated. Third, how attention plays a role in regulating emotion during sport performance warrants further investigation. Gross & Thompson (2007) proposed the modal model of emotion and inferred that the emotion generation process occurs in a particular sequence from situation, attention, and appraisal to response. Allocation of attention involves directing one’s attention towards or away from an emotional situation. Therefore, measures of attention could provide critical information for the mechanism in emotional and motivational regulation processes. And lastly, given that the differences in frontal alpha asymmetry between successful and unsuccessful putts following previously successful putts could also provide insight on the emotional and motivational regulation of the skilled golfers, future studies looking into this aspect are warranted.

## Conclusions

In conclusion, this study showed that skilled golfers successfully regulate their emotion and motivation by increasing relatively more left frontal activation during the last two seconds prior to putting execution when facing a performance failure, an emotionally provocative event commonly encountered by athletes. The study demonstrated the practical utility of the frontal alpha asymmetry for understanding the temporal dynamic of emotional and motivational regulation during sport performance.

##  Supplemental Information

10.7717/peerj.6777/supp-1 Dataset S1 Control analysis

10.7717/peerj.6777/supp-2 Dataset S2 Demography data

10.7717/peerj.6777/supp-3 Supplemental Information 3 After a failed putt, frontal alpha asymmetry were prior two seconds The left side of data is the original value, and the right side of data is frontal alpha asymmetry data. T1:-2- -1 s; T2:-1- 0 s; F3: left side of the frontal lobe; F4: right side of the frontal lobe. Success: successful trials which were putted into the hole; Unsuccessful: unsuccessful trials which were not putted into the hole.

## Additional Information and Declarations

### Data Availability

The following information was supplied regarding data availability: The raw data are provided in the Supplemental Files.
